# Ureteric Injury due to the Use of LigaSure

**DOI:** 10.1155/2013/989524

**Published:** 2013-09-19

**Authors:** Muazzam Tahir, William Gilkison

**Affiliations:** Taranaki Base Hospital, David Street, Westown, New Plymouth 4310, New Zealand

## Abstract

*Background*. LigaSure is a bipolar clamping device used in open and laparoscopic surgeries for producing haemostasis in vascular pedicles up to 7 mm in diameter (“*Covidien LigaSure technology: consistent, reliable, trusted vessel sealing,*” 2012). The use of LigaSure has made securing haemostasis and tissue dissection relatively easy especially in laparoscopic surgery; however, if not used with care it can cause damage to the surrounding structures through lateral spread of energy. *Case Report*. This case report discusses the induction of a thermal ureteral injury associated with the use of LigaSure. An 80-year-old gentleman was operated for bowel cancer. LigaSure was used for securing haemostasis and tissue dissection. Postoperatively, he was found to have damage to the right ureter secondary to lateral spread of energy from the jaws of LigaSure with high abdominal drain output. *Conclusion*. Judicious and careful use of electrosurgical devices should be done to prevent inadvertent damage to the surrounding structures. Early recognition and involvement of a urologist can prevent long-term complications.

## 1. Introduction

Haemorrhage is one of the most common complications during surgery. Adequate haemostasis is required for optimal intra- and postoperative results. A number of different haemostatic devices have been developed for this. One of these devices is LigaSure. It has been used in 4 million procedures since its launch in 1998 [1]. It is promoted as a safe alternative to other haemostatic devices.

## 2. Case Report

An 80-year-old male had an elective admission for an anterior resection for adenocarcinoma of the rectum. Initially, he had a laparoscopic approach but because of the poor bowel preparation (despite preoperative enemas) and the tumour being unable to be seen with either on-table rigid sigmoidoscopy or with the laparoscope, the operation was converted to an open procedure. During dissection, both ureters were clearly seen and reflected. The patient had a small pelvis with relatively bulky contents. An anastomosis was formed with a covering loop ileostomy. During both laparoscopic and open phases, LigaSure was used for dissection and to secure haemostasis. Postoperatively, he initially progressed well; however, his urinary catheter output gradually dropped, and his peritoneal drainage output increased over the first three days.

A sample of the peritoneal drain output had a high level of urea and the assumption was made that there has been a ureteric injury. A CT KUB was done which was inconclusive but raised the suspicion of ureteric injury by showing fluid in pelvis with density similar to that of the fluid in urinary bladder.

The patient was taken back to theatre where cystoscopy, bilateral retrograde ureterograms and insertion of JJ stent on the right side were done. He was found to have an injury to the right ureter with a defect in the medial portion of its lower third just below the pelvic brim. He was returned to HDU and had problems with fluid overload, type II NSTEMI, CHF, and fast atrial fibrillation over the next few days. Following all of this, he recovered well and was discharged home.

It is likely that he sustained a thermal injury to the right ureter from the use of LigaSure with later necrosis and perforation. This could be a result of direct damage to the ureter by holding it between the jaws of LigaSure during dissection but as both ureters were clearly identified and moved away, it is unlikely to be the cause of ureteric damage. More likely, it was the result of the conduction of thermal energy from tissues within the jaws of LigaSure during dissection close to the right ureter on its medial side.

## 3. Discussion

LigaSure is one of several methods used for securing haemostasis during surgery. Others include suture ligation, application of clips, conventional diathermy, and ultrasonic coagulation techniques. The development of LigaSure system was instigated by the need for a device that can be used for securing haemostasis and tissue dissection at the same time.

The LigaSure system consists of an electrical generator and a variety of instruments that can be used in a number of surgical procedures. The energy supplied can be varied as required to create a consistent seal with each application. LigaSure achieves haemostasis by vessel compression and by fusing the collagen and elastin in the walls of blood vessels by means of electrical bipolar energy [2]. Once the vessel obliteration is complete, a ringing sound informs the operator. There is a lateral spread of thermal energy which can damage the tissue but this is limited to 2 mm [3].

The use of electrical devices during surgery can damage the adjacent structures such as bowel, ureters, and nerves through lateral spread of thermal energy [4–6]. The lateral damage depends upon the type of instrument, duration of application to the tissue and power setting at which the instrument is used. A study conducted in Nottingham, UK [7], compared the lateral thermal spread using monopolar and bipolar diathermy, the harmonic scalpel, and the LigaSure. Porcine muscle was used for this purpose as it was believed to be the most appropriate substitute to human tissue.

 Each instrument was studied at three different power settings and was applied for three different durations. The temperatures were measured at the tip of the instrument and in the adjacent structures after every application. Monopolar diathermy generated the highest temperature at the tip of the instrument and in the adjacent tissues and therefore led to significant thermal spread laterally. The other three instruments were more or less the same in the lateral spread of thermal energy with LigaSure causing the least rise in temperature In conclusion, this study advised that careful use of the electrosurgical instruments (lower power settings and short application times) should be done to prevent inadvertent and unnecessary damage to the surrounding tissues [7].

An RCT conducted in Amsterdam [8] compared the effectiveness of LigaSure with other electrothermal and ultrasonic devices in the abdominal surgical procedures. No significant differences were found in the rate of complications between different devices; however, because of the small sample size it could not be concluded that one device was superior to the other, and much larger RCTs are required. 

As a rule, surgeons are not formally trained on energy-based devices and are not required to document knowledge regarding their safe use. They are not usually tested to check how safely are they using the devices [9]. A study done in Canada and USA assessed the knowledge of gastrointestinal surgeons regarding the use of the energy based devices. It was found that many surgeons lacked sound knowledge of safe use of electrical devices in surgery [9].

## 4. Conclusion

 All electrosurgical devices should be used with great care, at the lowest possible power settings, and for the shortest possible durations. Even with novel haemostatic devices, there is a potential for heat related damage to tissues near the target. Care should be taken to keep other tissues away from the tissues in the jaws of the instruments, but this is more difficult in the rigid confines of a male pelvis. 

 In this case, it was fortunate that the damage could be managed endoscopically, and the outlook for this injury is good.
